# Auxora versus standard of care for the treatment of severe or critical COVID-19 pneumonia: results from a randomized controlled trial

**DOI:** 10.1186/s13054-020-03220-x

**Published:** 2020-08-14

**Authors:** Joseph Miller, Charles Bruen, Michael Schnaus, Jeffrey Zhang, Sadia Ali, April Lind, Zachary Stoecker, Kenneth Stauderman, Sudarshan Hebbar

**Affiliations:** 1 Henry Ford Hospital System, Detroit, MI USA; 2 Regions Hospital, Health Partners, St. Paul, MN USA; 3 Methodist Hospital, Park Nicollet, St. Louis Park, MN USA; 4 University of Minnesota, Minneapolis, MN USA; 5 Princeton Pharmatech, Princeton, NJ USA; 6 CalciMedica, Inc., 505 Coast Blvd. South Suite 202, La Jolla, CA 92037 USA

**Keywords:** COVID-19 pneumonia, Calcium release-activated calcium channel inhibitors, CRAC channel inhibitors, Proinflammatory response, Pulmonary endothelium, Respiratory complications

## Abstract

**Background:**

Calcium release-activated calcium (CRAC) channel inhibitors stabilize the pulmonary endothelium and block proinflammatory cytokine release, potentially mitigating respiratory complications observed in patients with COVID-19. This study aimed to investigate the safety and efficacy of Auxora, a novel, intravenously administered CRAC channel inhibitor, in adults with severe or critical COVID-19 pneumonia.

**Methods:**

A randomized, controlled, open-label study of Auxora was conducted in adults with severe or critical COVID-19 pneumonia. Patients were randomized 2:1 to receive three doses of once-daily Auxora versus standard of care (SOC) alone. The primary objective was to assess the safety and tolerability of Auxora. Following FDA guidance, study enrollment was halted early to allow for transition to a randomized, blinded, placebo-controlled study.

**Results:**

In total, 17 patients with severe and three with critical COVID-19 pneumonia were randomized to Auxora and nine with severe and one with critical COVID-19 pneumonia to SOC. Similar proportions of patients receiving Auxora and SOC experienced ≥ 1 adverse event (75% versus 80%, respectively). Fewer patients receiving Auxora experienced serious adverse events versus SOC (30% versus 50%, respectively). Two patients (10%) receiving Auxora and two (20%) receiving SOC died during the 30 days after randomization. Among patients with severe COVID-19 pneumonia, the median time to recovery with Auxora was 5 days versus 12 days with SOC; the recovery rate ratio was 1.87 (95% CI, 0.72, 4.89). Invasive mechanical ventilation was needed in 18% of patients with severe COVID-19 pneumonia receiving Auxora versus 50% receiving SOC (absolute risk reduction = 32%; 95% CI, − 0.07, 0.71). Outcomes measured by an 8-point ordinal scale were significantly improved for patients receiving Auxora, especially for patients with a baseline PaO_2_/FiO_2_ = 101–200.

**Conclusions:**

Auxora demonstrated a favorable safety profile in patients with severe or critical COVID-19 pneumonia and improved outcomes in patients with severe COVID-19 pneumonia. These results, however, are limited by the open-label study design and small patient population resulting from the early cessation of enrollment in response to regulatory guidance. The impact of Auxora on respiratory complications in patients with severe COVID-19 pneumonia will be further assessed in a planned randomized, blinded, placebo-controlled study.

**Trial registration:**

ClinicalTrials.gov, NCT04345614. Submitted on 7 April 2020.

**Graphical abstract:**

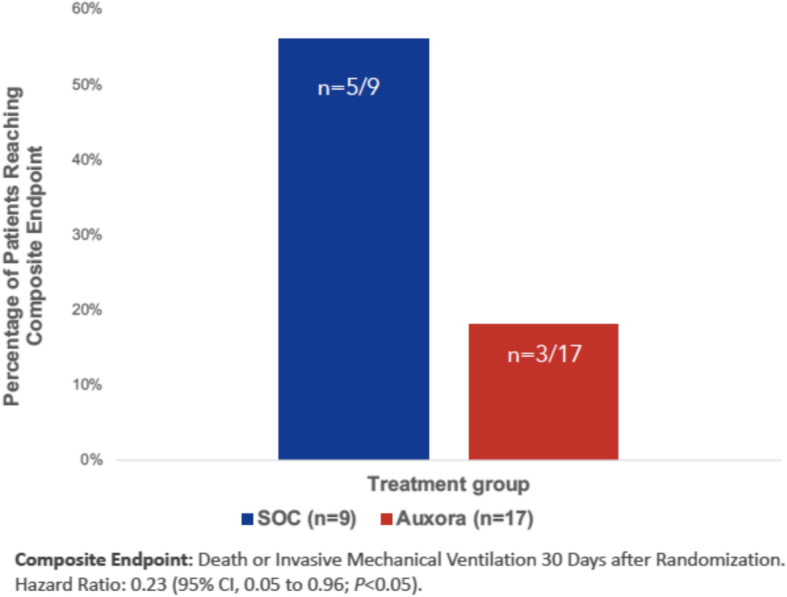

## Background

The novel severe acute respiratory syndrome coronavirus 2 (SARS-CoV-2), which causes the disease COVID-19, was first identified in December 2019 and designated a global pandemic by the World Health Organization in February 2020 [1]. The majority of COVID-19 cases are mild, but up to 20% of patients develop severe or critical pneumonia, manifested by hypoxemia or respiratory failure necessitating mechanical ventilation, respectively [1, 2]. COVID-19 pneumonia presents with a constellation of symptoms including fever, cough, and dyspnea, with infiltrates usually noted on lung imaging [1, 2]. While the pathophysiology for COVID-19 pneumonia remains under investigation, there is an increasing body of literature to suggest a multifactorial lung injury and the important role of a hyperinflammatory state in its development [3–5]. Among patients with COVID-19 pneumonia, viral infiltration has been shown to cause severe endothelial injury and diffuse alveolar damage [2–5]. Furthermore, there appears to be an increase in proinflammatory cytokines leading to additional lung injury [2–5]. Together, these factors contribute to clinical deterioration, the need for invasive mechanical ventilation, and, in a substantial proportion of patients, death [2–5].

Evidence suggests that calcium release-activated calcium (CRAC) channels play a role in inflammation-induced injury of pulmonary endothelial cells, resulting in loss of alveolar-capillary barrier function and extravasation of fluid into the alveoli [6–9]. CRAC channel activation is also linked to the production of proinflammatory cytokines associated with worsened outcomes in COVID-19 [7–11]. Thus, inhibition of CRAC channels may be beneficial in preserving pulmonary endothelial integrity, reducing proinflammatory cytokine levels, and improving oxygenation in patients with COVID-19 pneumonia [8, 11–14].

CM4620 is a potent and selective CRAC channel inhibitor [11–13, 15]. Preclinical work has demonstrated that in acute inflammatory conditions, CM4620 reduces inflammatory signals in the lung, protects tissues from calcium-induced damage, and lowers serum and pulmonary proinflammatory cytokine levels [7, 9, 11, 12]. Auxora, the novel intravenously administered nanoemulsion formulation of CM4620, rapidly distributes to the lungs and blocks CRAC channel-dependent cytokine release within hours of its administration [15]. Based on these data and rationale, Auxora was investigated in patients with severe or critical COVID-19 pneumonia. The interim analyses presented here describe the safety of Auxora for all patients enrolled in the study; the efficacy analysis is limited to those with severe COVID-19 pneumonia.

## Methods

### Patient selection

This phase 2, randomized, controlled, open-label study was conducted across three centers in the USA (ClinicalTrials.gov number NCT04345614). Patient enrolment took place from April 8, 2020, to May 13, 2020. Eligible patients were adults with a diagnosis of COVID-19 determined by reverse transcription polymerase chain reaction and pneumonia documented by chest imaging. In addition, patients were required to have ≥ 1 symptom consistent with COVID-19, such as fever, cough, sore throat, malaise, headache, muscle pain, dyspnea, confusion, or respiratory distress, and ≥ 1 clinical sign suggesting respiratory compromise, such as respiratory rate ≥ 30 breaths per minute, heart rate ≥ 125 bpm, SpO_2_ < 93% on room air or requiring > 2 L oxygen by nasal cannula to maintain SpO_2_ ≥ 93%, or PaO_2_/FiO_2_ < 300, imputed from pulse oximetry or determined by arterial blood gas.

### Study design

The initial study design included enrolment of 60 patients receiving low-flow supplemental oxygen at screening into arm A (severe COVID-19 pneumonia) and 60 patients receiving high-flow supplemental oxygen through a high-flow nasal cannula at screening into arm B (critical COVID-19 pneumonia). In both arms, patients were randomly assigned in a 2:1 ratio to receive Auxora plus standard of care or standard of care alone; randomization was not center specific. Auxora was administered on three consecutive days as a 4-h continuous intravenous infusion. The initial dose was 2.0 mg/kg (max 250 mg), and subsequent doses were 1.6 mg/kg (max 200 mg) at 24 and 48 h. All patients received local standard of care, including antiviral agents, but investigational therapies and immunosuppressive medications were not permitted. At the discretion of the site investigators, patients treated with either Auxora or standard of care alone were able to receive convalescent plasma if they required invasive mechanical ventilation.

After admission, patients were assessed daily for the first 10 days and then every 48 h until day 28 or discharge, whichever occurred first. On day 30, all patients were assessed for mortality. Discharged patients were contacted by phone. All adverse events (AEs) and serious adverse events (SAEs) were recorded during hospitalization. The SpO_2_ and FiO_2_ at the time of the study visit and the lowest SpO_2_/FiO_2_ ratio documented over the previous 24 h were recorded daily. The patient’s clinical status was also evaluated daily by assessing if the patient was alive and required invasive mechanical ventilation or extracorporeal membrane oxygenation (ECMO), high-flow supplemental oxygen or non-invasive ventilation, low-flow supplemental oxygen, or other ongoing medical care in the previous 24 h. The need for continued supplemental oxygen was also assessed at the time of discharge.

The trial protocol was approved by an institutional review board at each site and was overseen by an independent safety review committee (ISRC). Informed consent was obtained from either the patient or from the patient’s legally authorized representative if the patient was unable to provide consent. The ISRC was scheduled to perform a review for each arm after the first 12 patients were dosed with Auxora and then again after 24 patients were dosed. The analysis plan called for a separate evaluation of the safety and efficacy of the two arms as enrolment rates were expected to differ. The ISRC conducted an initial review on May 3, 2020, after the first 12 patients in arm A were dosed with Auxora. At that time, six patients had received standard of care in arm A. The ISRC recommended continuing the trial without changes. The US Food and Drug Administration (FDA) was sent the interim efficacy data presented here in response to questions about the ISRC review. The FDA provided guidance on May 12, 2020, to limit further enrolment in the open-label study and transition to a randomized, blinded, placebo-controlled study, and as such, both arms A and B ceased further enrolment.

### Statistical analysis

All analyses were conducted in the intention-to-treat population. The primary objective was to determine the safety and tolerability of Auxora for patients with severe and critical COVID-19 pneumonia. The incidence, intensity, and relationship of AEs and SAEs and the development of laboratory abnormalities that were clinically significant and required intervention were assessed. Mortality at day 30 was evaluated as a safety outcome. For the purpose of safety and outcome analyses, patients from arms A and B were analyzed together, comparing treatments with Auxora and standard of care with standard of care alone.

Efficacy outcome measures included recovery rate defined as the first day the patient satisfied criterion 6, 7, or 8 of the 8-point ordinal scale (Table 1). Additional efficacy outcome measures included the change in the 8-point ordinal scale over time, the proportion of patients requiring invasive mechanical ventilation, and a composite outcome of death or invasive mechanical ventilation.

**Table 1:** Eight-point ordinal scale

Scale	Description
1	Death
2	Hospitalized, requiring invasive mechanical ventilation or ECMO
3	Hospitalized, requiring non-invasive mechanical ventilation or high-flow supplemental oxygen
4	Hospitalized, requiring low-flow supplemental oxygen
5	Hospitalized, not requiring supplemental oxygen but requiring ongoing medical care
6	Hospitalized, not requiring supplemental oxygen or ongoing medical care
7	Discharged, requiring supplemental oxygen
8	Discharged, not requiring supplemental oxygen

Efficacy outcome measured with the 8-point ordinal scale included recovery rate defined as the first day the patient satisfied criterion 6, 7, or 8 and change in the 8-point ordinal scale over time

*ECMO* extracorporeal membrane oxygenation

Pre-specified efficacy analyses were performed on enrolled patients with severe COVID-19 pneumonia (arm A) and in 3 subgroups of arm A according to their baseline PaO_2_/FiO_2_ (1–100, 101–200, or ≥ 201). Baseline PaO_2_/FiO_2_ was defined as the lowest value in the 24 h prior to screening. The PaO_2_ was imputed from the SpO_2_ using a published table based on Ellis’s inversion of the Severinghaus equation [16, 17].

## Results

### Patients

**Fig. 1:**
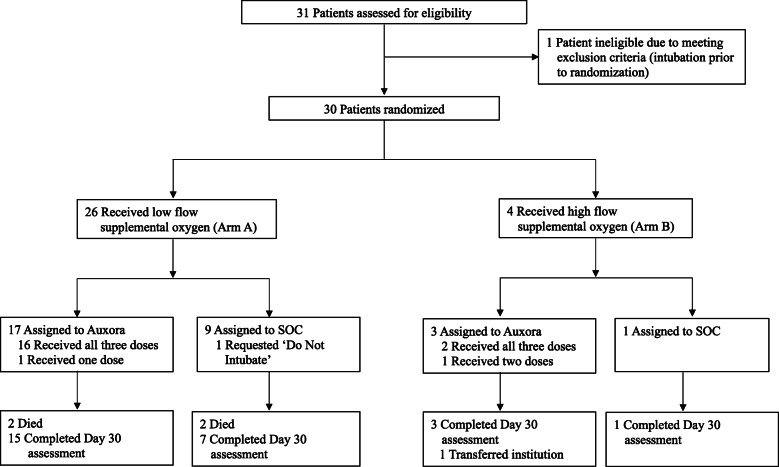
Patient enrolment and randomization. One patient in arm A received only 1 Auxora dose due to rapid improvement and early discharge, and 1 patient in arm B refused the third dose of Auxora. One patient in arm B, who received all 3 doses, was transferred after 120 h to another institution. In the standard of care group of arm A, 1 patient withdrew from the study at 96 h after being made do not intubate (DNI) because of declining respiratory status

At the time of cessation, 30 patients had been enrolled in the study. Of the 26 patients in arm A, 17 were randomized to treatment with Auxora and nine to standard of care alone. Four patients were enrolled in arm B: three to treatment with Auxora and one to standard of care alone (Fig. 1). Across both arms, 18 patients (90%) received three doses of Auxora as assigned. One patient in arm A received only one Auxora dose due to rapid improvement and early discharge. One patient in arm B refused the third dose of Auxora. One patient in arm B, who received all three doses, was transferred after 120 h to another institution; their outcome was followed by the initial study team. In the standard of care group of arm A, one patient withdrew from the study at 96 h after being made do not intubate (DNI) because of declining respiratory status. This patient was not included in the intubation analysis (Fig. 1). All patients who did not die in the hospital completed the day 30 assessment.

Baseline demographics were balanced across the Auxora and standard of care groups in arm A (Table 2), but more patients in the Auxora group had diabetes (47%) than those in the standard of care group (22%). The median time (min, max) from symptom onset to randomization was 9 (4, 34) days in the Auxora group and 7 (4, 11) days in the standard of care group. The baseline mean imputed PaO_2_/FiO_2_ was 178 ± 74 in the Auxora group and 168 ± 78 in the standard of care group. In arm B, baseline characteristics were more variable due to the small sample size (Table 1). Individual patient listings for arm A are presented in the supplementary appendix Figure S1.

**Table 2:** Baseline demographics and clinical characteristics

	Arm A*		Arm B^†^	
	Auxora (***n*** = 17)	SOC (***n*** = 9)	Auxora (***n*** = 3)	SOC (***n*** = 1)
Age, years (mean ± SD)	59 ± 12	61 ± 13	64 ± 14	36 ± NA
Median BMI, kg/m^2^ (min, max)	30 (25, 79)	30 (23, 49)	23 (18, 36)	34
Male sex, *n* (%)	7 (41)	5 (56)	1 (33)	1 (100)
Ethnicity, *n* (%)				
White	8 (47)	5 (56)	1 (33)	0
Black or African American	7 (41)	3 (33)	1 (33)	1 (100)
Asian	0	1 (11)	1 (33)	0
Others/multiple	2 (12)	0	0	0
Hispanic or Latino, *n* (%)	2 (12)	1 (11)	0	0
Diabetes, *n* (%)	8 (47)	2 (22)	2 (67)	0 (0)
Hypertension, *n* (%)	8 (47)	4 (44)	2 (67)	0 (0)
Median time from the onset of symptoms to randomization, days (min, max)	9 (4, 34)	7 (4, 11)	11 (8, 17)	13
Ferritin, ng/mL (mean ± SD)	709 ± 553	772 ± 742	1776 ± 722	2151 ± NA
CRP, mg/dL (mean ± SD)	10 ± 7	12 ± 6	14 ± 11	9 ± NA
PaO_2_/FiO_2_ (mean ± SD)	178 ± 74	168 ± 78	106 ± 45	87 ± NA
PaO_2_/FiO_2_ ≥ 201	7 (41)	3 (33)	0	0
PaO_2_/FiO_2_ 101–200	6 (35)	4 (44)	2 (67)	0
PaO_2_/FiO_2_ ≤ 100	4 (24)	2 (22)	1 (33)	1 (100)
PaO_2_/FiO_2_ 101–200 ferritin ng/mL (mean ± SD)	867 ± 712	910 ± 1090	1637 ± 963	NA
PaO_2_/FiO_2_ 101–200 CRP mg/dL (mean ± SD)	11 ± 5	13 ± 9	16 ± 15	NA

Baseline demographics were balanced across the Auxora and standard of care groups in arm A. In arm B, baseline characteristics were more variable due to the small sample size

*NA* not available, *SOC* standard of care

*Patients in arm A included those who were receiving low-flow supplemental oxygen at screening and were defined by regulatory guidance as having severe COVID-19 pneumonia

^†^Patients in arm B were defined by regulatory guidelines as having critical COVID-19 pneumonia

Fewer patients randomized to Auxora versus standard of care received steroids (47% versus 78%) or remdesivir (18% versus 33%) during the course of the study. Convalescent plasma was administered to 2 patients randomized to Auxora, while it was administered in all 4 patients randomized to standard of care who needed mechanical ventilation.

### Safety outcomes

Across both arms, 15 patients (75%) receiving Auxora had ≥ 1 AE and six patients (30%) had ≥ 1 SAE. Site investigators judged three AEs, each occurring in three different patients, as being related to the administration of Auxora: an episode of itching, an increase in alkaline phosphate, and a rash. They were all considered mild by the investigators and resolved. None of the reported SAEs was determined to be related to the administration of Auxora. Among patients receiving standard of care, eight (80%) had ≥ 1 AE and five (50%) had ≥ 1 SAE. There was no difference in AEs related to infections in the Auxora group when compared to standard of care (30% in each group).

Two patients (10%) treated with Auxora and two patients (20%) receiving standard of care died while hospitalized between 10 and 17 days after randomization (supplementary appendix Figure S1). There were no deaths in the 30 days after randomization for patients who were discharged from the hospital. Both patients in the Auxora group and one patient in the standard of care group died while receiving invasive mechanical ventilation. The other patient receiving standard of care who died had been made DNI.

### Efficacy outcomes

**Fig. 2:**
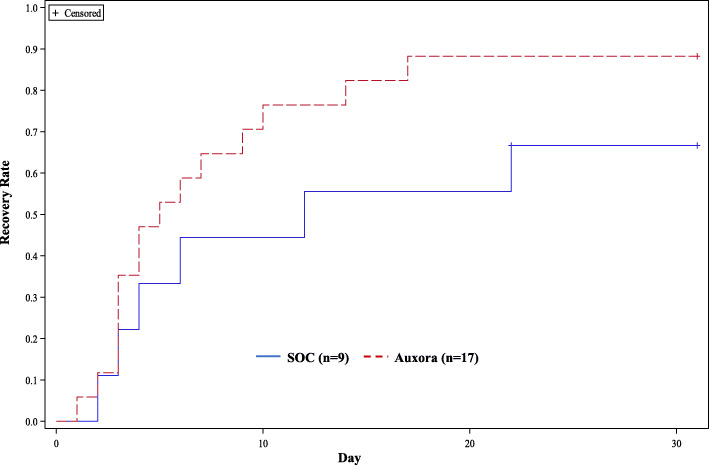
Recovery rate among patients with severe COVID-19 pneumonia. Recovery rate defined as the first day the patient satisfied criterion 6, 7, or 8 of the 8-point ordinal scale. Patients receiving Auxora had a shorter median time to recovery (5 days) than patients treated with standard of care (12 days); recovery rate ratio was 1.87 (95% CI, 0.72 to 4.89). Patients with severe COVID-19 pneumonia were receiving low-flow supplemental oxygen (arm A)

**Fig. 3:**
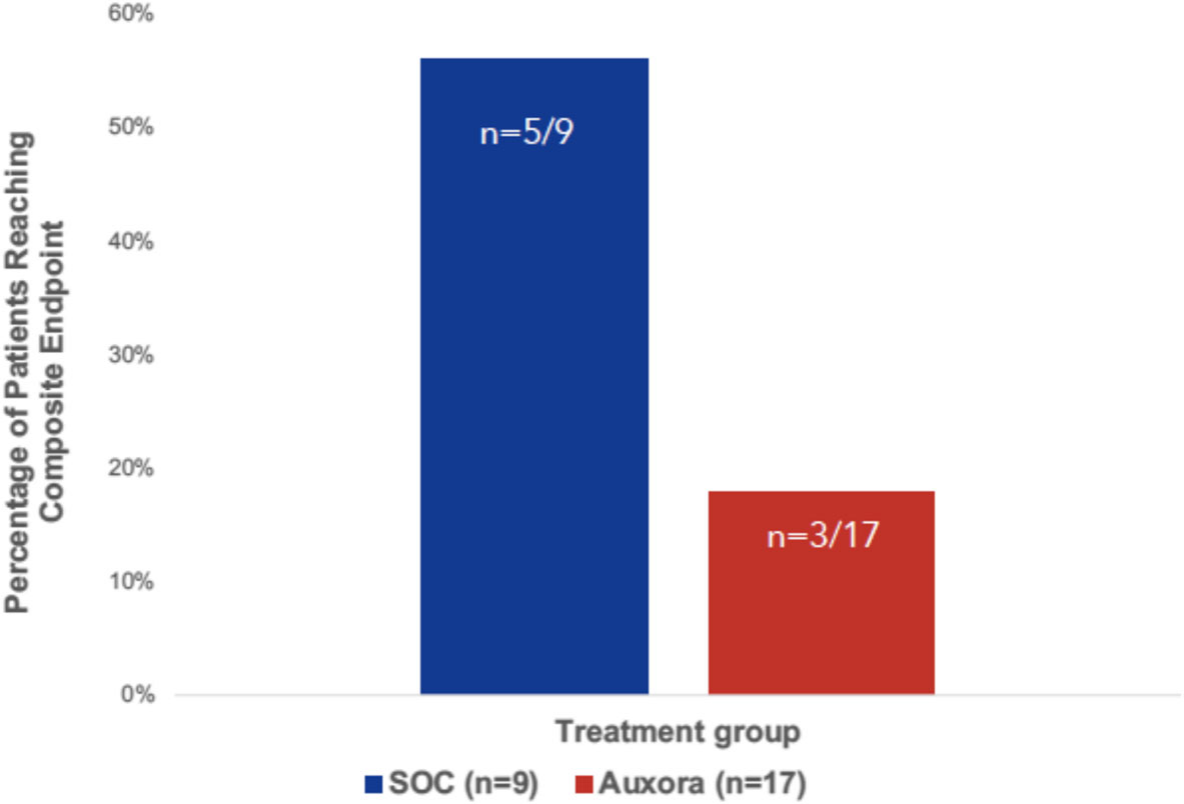
Percentage of patients with severe COVID-19 pneumonia reaching a composite endpoint. The composite endpoint was defined as needing invasive mechanical ventilation or death in the 30 days after randomization. Hazard ratio was 0.23 (95% CI, 0.05 to 0.96; *P* < 0.05). Patients with severe COVID-19 pneumonia were receiving low-flow supplemental oxygen (arm A)

Patients with severe COVID-19 pneumonia (arm A) treated with Auxora had a shorter median time to recovery (5 days) than patients treated with standard of care (12 days); the recovery rate ratio was 1.87 (95% confidence interval [CI], 0.72 to 4.89; Fig. 2). In addition, three of 17 patients treated with Auxora (18%) were intubated compared to four of eight (50%) assigned to standard of care (95% CI, − 0.07 to 0.71). The reduction was most pronounced in patients with a baseline PaO_2_/FiO_2_ between 101 and 200, in which only one of six patients (17%) treated with Auxora required intubation compared to three of four patients (75%) assigned to standard of care. No patients receiving Auxora or standard of care with a baseline PaO_2_/FiO_2_ > 200 required invasive mechanical ventilation. A composite endpoint of death or invasive mechanical ventilation occurred less frequently in patients treated with Auxora (18%) compared to those assigned to standard of care (56%) with a hazard ratio of 0.23 (95% CI, 0.05 to 0.96; *P* < 0.05; Fig. 3).

**Fig. 4:**
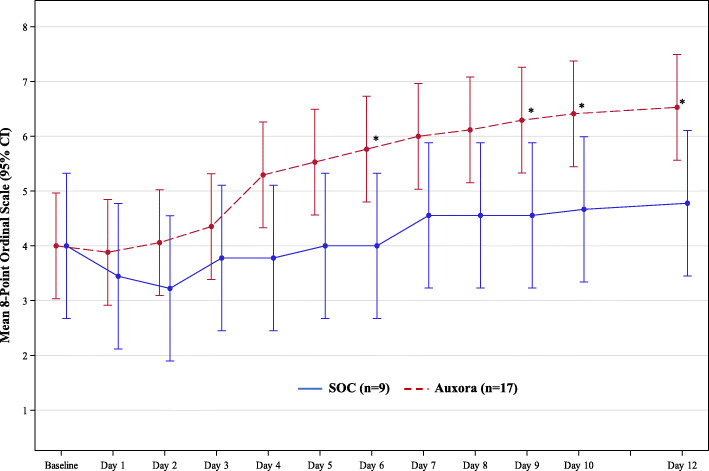
Eight-point ordinal scale over time in patients with severe COVID-19 pneumonia. The mean difference was statistically significant for Auxora (*n* = 17) when compared with standard of care (*n* = 9) at day 6 and days 9 through 12 (*P < 0.05 versus standard of care). Patients with severe COVID-19 pneumonia were receiving low-flow supplemental oxygen (arm A)

**Fig. 5:**
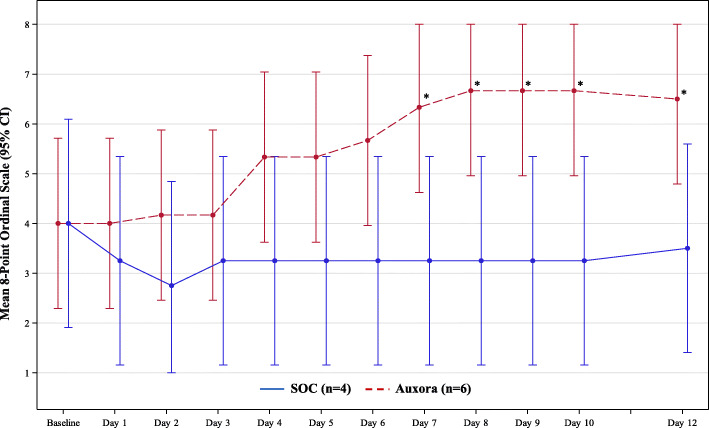
Eight-point ordinal scale in patients with severe COVID-19 pneumonia with PaO_2_/FiO_2_ between 101 and 200. The mean difference was statistically significant from day 7 to day 12 for patients receiving Auxora (*n* = 6) compared with those receiving standard of care (*n* = 4; *P < 0.05 versus standard of care). Patients with severe COVID-19 pneumonia were receiving low-flow supplemental oxygen (arm A)

Clinical improvement, as measured by the mean of an 8-point ordinal scale, was greater in the Auxora group starting at day 4, reaching statistical significance on day 6, and remained significant from day 9 to day 12 (*P* < 0.05; Fig. 4). On day 4, the odds ratio for clinical deterioration on the 8-point ordinal scale for the Auxora group compared to the standard of care group was 0.21 (95% CI, 0.04 to 0.098; *P* < 0.05). The clinical improvement was most pronounced in patients with a baseline PaO_2_/FiO_2_ between 101 and 200, with the difference in means reaching statistical significance at day 7; this was maintained through day 12 (*P* < 0.05; Fig. 5).

## Discussion

In this phase 2, open-label, randomized, multicenter study of patients with severe or critical COVID-19 pneumonia, Auxora, a novel, intravenously administered CRAC channel inhibitor, demonstrated a potential therapeutic benefit in mitigating the respiratory complications of COVID-19. At the recommendation of the US FDA, this study was halted prior to completion of the originally planned 120 patients in order to transition to a randomized, placebo-controlled, double-bind study.

CRAC channel activation in the pulmonary endothelium is linked to the breakdown of the alveolar-capillary barrier [7–9]. CRAC channel activation also initiates the production and release of proinflammatory cytokines from immune cells [10, 11]. The resulting development of pulmonary edema, hypoxemia, and ultimately ARDS contributes to the significant morbidity and mortality seen in COVID-19 pneumonia, particularly in those who eventually require invasive mechanical ventilation [14, 18]. As demonstrated in animal models, inhibition of CRAC channels stabilizes pulmonary endothelial cells, blocks the release of proinflammatory cytokines, and decreases vascular inflammation and permeability [7, 9, 14]. Given the direct effects on the pulmonary endothelium and the indirect effects on proinflammatory cytokine production, Auxora may be an attractive therapy for the management of patients with severe COVID-19 pneumonia [7–9]. Auxora has also demonstrated rapid distribution to the lungs, resulting in a fast onset of action that is reversible in 24 to 48 h (unpublished observations).

The data available at the time of study termination indicate an encouraging safety profile for Auxora, with no increase in the proportion of patients experiencing AEs or SAEs when compared with standard of care. Furthermore, while the sample size was small, patients receiving Auxora appeared to have a more favorable clinical course than patients receiving standard of care as reflected by the rapid time to recovery; the decreased need for steroids, remdesivir, convalescent plasma, or invasive mechanical ventilation; and greater improvement in clinical outcomes as documented by the difference in 8-point ordinal scale. Analysis of the ordinal scale over time also suggested greater odds of improvement in patients treated with Auxora beginning on day 4, with the most pronounced clinical benefit in patients with a PaO_2_/FiO_2_ ratio between 101 and 200. These efficacy signals will need to be confirmed in a larger, double-blind, placebo-controlled study.

The ideal timing and duration of intervention in the course of COVID-19 pneumonia remain unknown [19]. There are some concerns that premature immunomodulation may inhibit host antiviral immunity and delay viral clearance, while delaying immunomodulation may prove futile if acute pulmonary injury is advanced. The PaO_2_/FiO_2_ ratio, imputed from the SpO_2_/FiO_2_, could serve as a simple means of determining both the optimal timing of intervention and the patients most likely to benefit [16, 17]; it should be incorporated into other studies of therapies for COVID-19. Currently, patients are only being categorized by the receipt of either low-flow supplemental oxygen or high-flow supplemental oxygen, but this approach may not capture the severity of lung injury, may mask patients who are likely to respond to treatment, and is limited by substantial inter-institution variations in practice.

Our findings, and those from recent remdesivir and RECOVERY trials, raise consideration for a two-pronged approach for the treatment of COVID-19 pneumonia [19, 20]. Preliminary results of remdesivir for the treatment of COVID-19 demonstrated reduced time to recovery, but treatment alone with an antiviral therapy is unlikely to be sufficient in improving outcomes [19]. It may be possible, however, to improve patient outcomes by combining an antiviral treatment with immunomodulation to address the inflammatory response. Results from the RECOVERY trial demonstrated that after up to 10 days of receiving oral or intravenous dexamethasone once daily, the 28-day mortality was lower than that in the usual care group among patients receiving invasive mechanical ventilation (29.3% versus 41.4%, respectively) and among those receiving oxygen without invasive mechanical ventilation (23.3% versus 26.2%, respectively) [20]. The 28-day mortality rates in the RECOVERY trial for patients receiving low-flow supplemental oxygen versus high-flow supplemental oxygen were not presented. Finally, there was a non-significant decrease in a composite outcome of death or invasive mechanical ventilation among patients not receiving invasive mechanical ventilation at randomization who were treated with dexamethasone compared to those treated with usual care (25.6% versus 27.3%, respectively) [20]. As Auxora is associated with a rapid onset of action and a rapid cessation of action, it appears to work quickly to reduce proinflammatory cytokines while preserving pulmonary endothelial integrity, without excessive immunosuppression [15] but further research is needed in patients with severe COVID-19 pneumonia. Coupled with the low 28-day mortality rate (10%) presented here, Auxora may allow for improved patient outcomes when used in combination with dexamethasone. Additional clinical trials are needed to understand the effect of these combinations of therapy.

The interpretation of the results of this study is limited by the open-label design and small sample size. Additionally, the significant imbalances in age and medical comorbidities between the three patients receiving Auxora and one patient receiving standard of care in arm B prevented a meaningful direct comparison between the two groups with critical COVID-19 pneumonia. It was observed that the proportion of patients treated with Auxora who had diabetes, a co-morbidity associated with poorer outcomes in COVID-19, was double that for patients receiving standard of care alone. Manufacturing and administration of a placebo were sacrificed given the need to initiate the study rapidly during the global pandemic with associated constraints on healthcare resources. These constraints, including adequate personal protective equipment, also limited the ability of research teams to obtain research-specific cytokine levels, which if obtained, may have further supported the mechanisms of Auxora.

## Conclusions

In this preliminary, phase 2 study of the novel CRAC inhibitor, Auxora, the observed favorable safety profile and efficacy signals when compared to standard of care support the need for further investigation in a large, double-blind, placebo-controlled trial in patients with severe COVID-19 pneumonia. In addition, these results suggest the potential for the clinical development of Auxora for the treatment of other etiologies of acute respiratory distress syndrome. A randomized, placebo-controlled, double-blind study will soon be underway to test the efficacy of Auxora in combination with local standard of care, likely remdesivir and/or dexamethasone, for the management of patients with severe COVID-19 pneumonia.

## Supplementary information

**Additional file 1.** Supplementary Appendix: Auxora versus standard of care for the treatment of severe or critical COVID-19 pneumonia: results from a randomized controlled trial.

## Data Availability

The datasets generated and/or analyzed during the current study are not publicly available due to the clinical study report being finalized but will be available from the corresponding author on reasonable request at a later time.

## Abbreviations

AEs: Adverse events
CRAC: Calcium release-activated calcium
DNI: Do not intubate
ECMO: Extracorporeal membrane oxygenation
FDA: Food and Drug Administration
ISRC: Independent Safety Review Committee
SAEs: Serious adverse events
SARS-CoV-2: Severe acute respiratory syndrome coronavirus 2
