# Supplementary material for: Auxora versus standard of care for the treatment of severe or critical COVID-19 pneumonia: results from a randomized controlled trial
Source: Crit Care. 2020 Aug 14;24:502. doi: 10.1186/s13054-020-03220-x (PMC7427272; doi:10.1186/s13054-020-03220-x)
Supplement: Supplementary file 1 — Additional file 1. Supplementary Appendix: Auxora versus standard of care for the treatment of severe or critical COVID-19 pneumonia: results from a randomized controlled trial. [file 13054_2020_3220_MOESM1_ESM.docx]

**Supplementary Appendix for the Manuscript Entitled**

**Auxora for the Treatment of Severe or Critical COVID-19 Pneumonia: Results from a Randomized Controlled Trial**

Table of Contents

[Study Team Members 2](#_Toc43233680)

[Supplemental Figure 3](#_Toc43233681)

# **Study Team Members**

We acknowledge and thank the members of the Regions Hospital Critical Care Research Center and the Henry Ford Hospital System for their participation in this clinical trial.

**Regions Hospital Critical Care Research Center Team Members**Nell Adams
Lydia Hamel
Jenny Koops
Sarah Matzdorf
Marissa Peterson
Paula Rupp
Sandi Wewerka
Kyra Wicklund

**Henry Ford Hospital System Team Members**

Mayur Ramesh, MD

Guneet Ahluwalia, DO

Kathleen Wilson, RN

Timothy Asmar

Margaret Beyer

Rebecca Bussa

Kaleem Chaudhry

Lakeeda Johnson

Jo-Ann Rammal

Jacob Ross

# **Supplemental Figure**

**Supplemental Figure S1. Overview of Patients with Severe COVID-19 Pneumonia (Low Flow Supplemental Oxygen; Arm A**). Individual patient listings. Dark shading of green (SOC) and blue (Auxora) represents treatment failure as indicated by the need for intubation (Int). The red boxes represent the point at which a patient died. One patient in the SOC group issued a Do Not Intubate (DNI) order.
